# The Guiding Symptoms of Our Materia Medica, Vol. VIII

**Published:** 1891-03

**Authors:** 


THE GUIDING SYMPTOMS OF OUR MATERIA MEDICA. By C. Hering, M. D. Volume Eighth. Philadelphia. Published by the estate of Constantine Hering: F. A. Davis, 1231 Filbert Street.

We have so often spoken of and recommended this work to our readers, both in our pages and in private letters, that it seems superfluous to speak of it again. Yet the book is so valuable, it is so necessary to every one making truly homœopathic prescriptions, that we are impelled to refer to it again, and urge every one of our readers to purchase it. The present volume includes the remedies from Natrum-phosphoricum to Pulsatilla, and they are arranged in a manner uniform with the preceding volume. W. M. J.

